# Supplementary material for: Focused learning by antibody language models using preferential masking of non-templated regions
Source: bioRxiv. 2024 Oct 28:2024.10.23.619908. Preprint. [Version 1] doi: 10.1101/2024.10.23.619908 (PMC11565838; doi:10.1101/2024.10.23.619908)
Supplement: 1 [file NIHPP2024.10.23.619908V1-supplement-1.pdf]

bioRxiv preprint doi: https://doi.org/10.1101/2024.10.23.619908; this version posted October 28, 2024. The copyright holder for this preprint (which was not certified by peer review) is the author/funder, who has granted bioRxiv a license to display the preprint in perpetuity. It is made available under aCC-BY 4.0 International license.

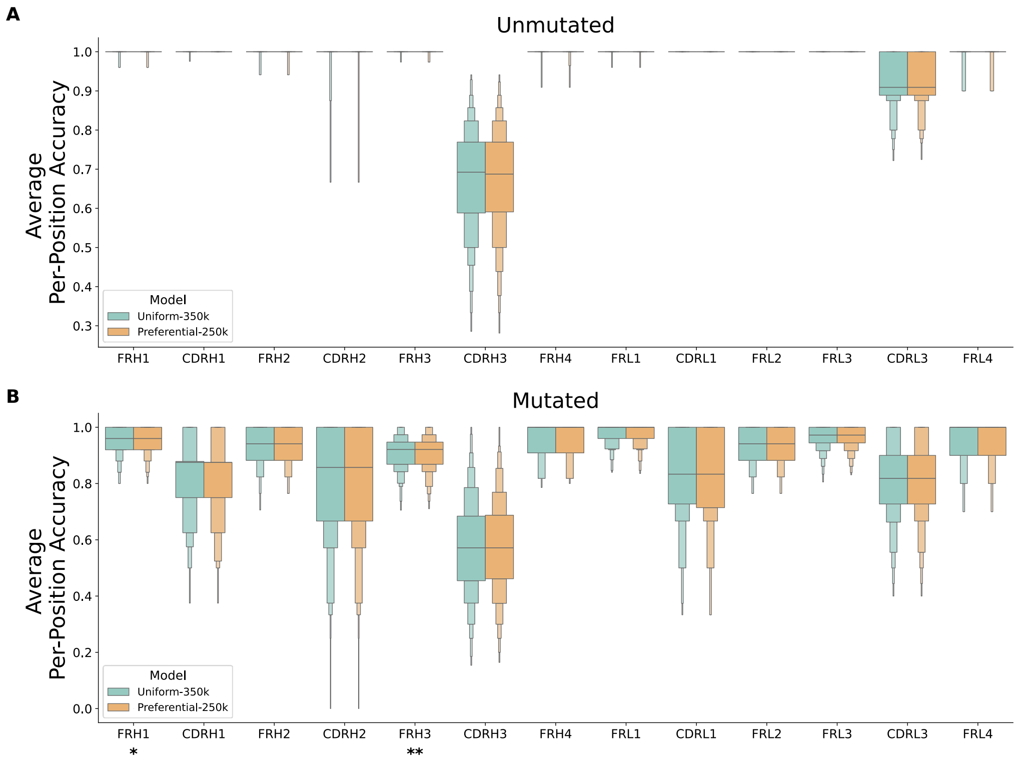

Figure S1. Per-position residue prediction accuracy. For 1000 unmutated (***A***) and mutated (***B***) test sequences, each residue was iteratively masked and predicted by both models (Uniform-350k and Preferential-250k). Mean prediction accuracy is plotted for each FR and CDR. Statistical significance for each region was calculated using a two-sided paired t-test with Bonferroni correction for multiple testing (14 regions).

bioRxiv preprint doi: https://doi.org/10.1101/2024.10.23.619908; this version posted October 28, 2024. The copyright holder for this preprint (which was not certified by peer review) is the author/funder, who has granted bioRxiv a license to display the preprint in perpetuity. It is made available under aCC-BY 4.0 International license.

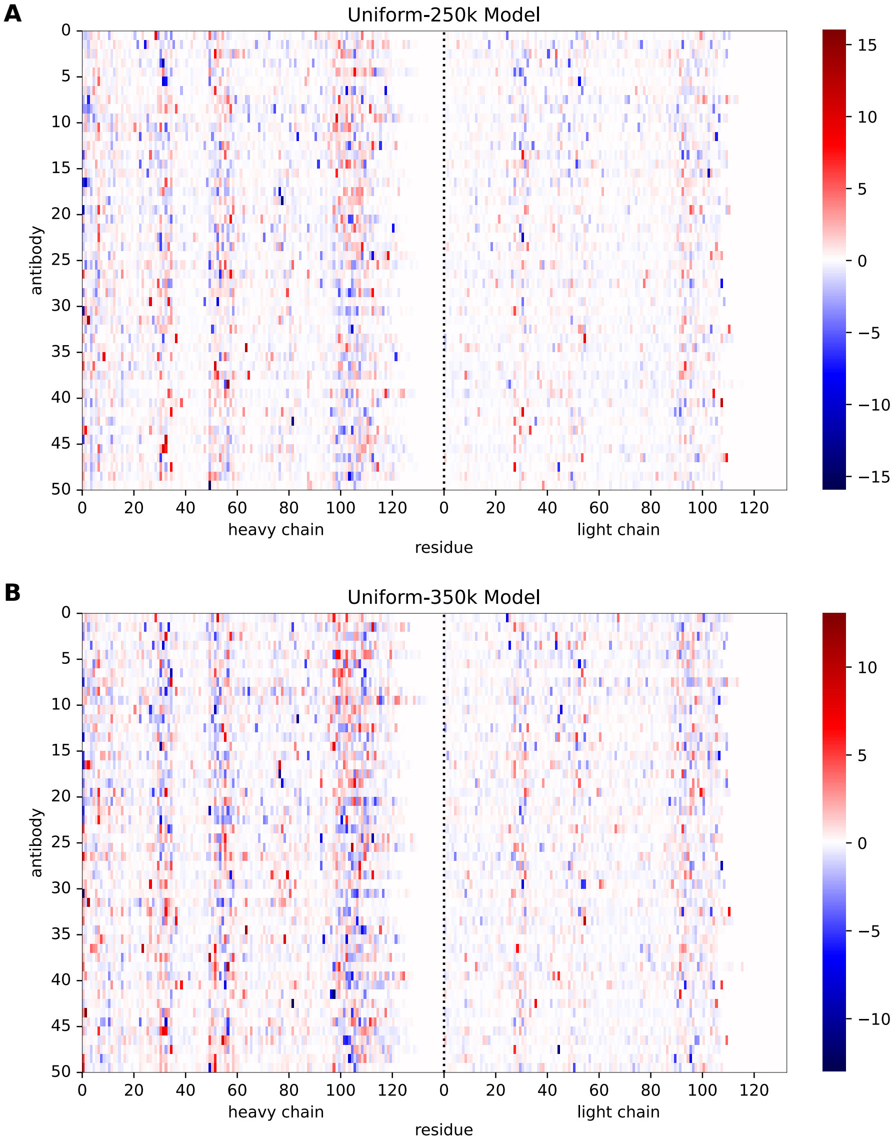

Figure S2. AttCAT analysis on the Uniform-250k and Uniform-350k CoV specificity classifier models. Normalized AttCAT impact scores with respect to the correct label class for the same 50 systematically chosen test sequences for the CoV specificity classifiers trained on the Uniform-250k (***A***) and Uniform-350k (***B***) base models. Sequences are sorted in ascending order by average prediction probability across the classifiers trained on all 3 base models.
